# Comparative Study of Elevated CA19-9 Levels in Non-Gastrointestinal Tumors Patients: Evaluation of Different Immunoassay Methods and Analysis of Potential Interfering Factors

**DOI:** 10.3390/biomedicines13061386

**Published:** 2025-06-05

**Authors:** Yangyang Liu, Wenxuan Li, Shaoxi Tang, Ruihao Wu, Yumin Wang, Fanggui Shao

**Affiliations:** 1 Central Laboratory, The First Affiliated Hospital of Wenzhou Medical University, Wenzhou 325000, China; liuyangyang@wmu.edu.cn; 2 Department of Laboratory Medicine, The First Affiliated Hospital of Wenzhou Medical University, Wenzhou 325000, China; l15924783264@163.com (W.L.); 15957816581@163.com (S.T.); wrhfulao1986@163.com (R.W.); 3 The First Clinical Medical School of Wenzhou Medical University, Wenzhou 325000, China; 4 Key Laboratory of Clinical Laboratory Diagnosis and Translational Research of Zhejiang Province, Department of Clinical Laboratory, The First Affiliated Hospital of Wenzhou Medical University, Wenzhou 325000, China

**Keywords:** CA19-9, non-gastrointestinal tumors, heterophilic antibody, concordance analysis

## Abstract

**Objectives**: CA19-9 elevation in non-gastrointestinal tumor patients may be influenced by various non-tumor factors, which poses challenges for clinical diagnosis. This study aims to assess the consistency between initial elevated CA19-9 levels detected by the ARCHITECT/Alinity i system (Abbott Diagnostics) and subsequent retesting using the Elecsys CA19-9 assay (Roche Diagnostics) in 5372 non-gastrointestinal tumor patients, and to explore potential factors contributing to CA19-9 non-specific elevation. **Methods**: Bland-Altman and Passing-Bablok analyses were used to assess the agreement between the two assays. Nonparametric Spearman and Pearson’s chi-square tests were used to assess the correlation between CA19-9 and different clinical comorbidities/antigen concentration strata and to compare the categorization by age/disease, respectively. **Results**: Bland–Altman and Passing–Bablok regression analyses revealed that the CA19-9 test results from Abbott and Roche platforms show significant systematic bias and weak correlation, making the two methods not directly interchangeable. After excluding common confounders, the study focused on heterophilic antibodies (HAs) as target. Blood samples were treated with a commercial blocking agent demonstrated alignment with baseline Elecsys CA19-9 results but differed significantly from initial ARCHITECT/Alinity i measurements. Furthermore, non-specific CA19-9 elevation was also associated with comorbidities such as diabetes mellitus, pulmonary infections, breast nodules, uterine leiomyoma, and its incidence increased with age. **Conclusions**: The study highlights the need to consider potential interferences and underlying disorders when results conflict with clinical diagnoses. Method-specific validation and comprehensive clinical correlation are crucial for accurate interpretation of CA19-9 levels to prevent misdiagnosis and ensure appropriate patient management.

## 1. Introduction

Carbohydrate antigen 19-9 (CA19-9) has emerged as a key biomarker in clinical oncology, maintaining its diagnostic significance through integration into multi-marker panels alongside AFP, CEA, CA125, and CA153 for routine surveillance [1,2,3,4]. This sialyl-Lewis (a) antigen is typically present in trace amounts (<37 U/mL) in healthy populations but exhibits dynamic elevation patterns across various pathological conditions, including benign hepatobiliary diseases and malignancies, such as acute cholangitis, pancreatic cancer, colorectal cancer, gastric cancer and so on [5,6,7].

Contemporary detection platforms include chemiluminescent immunoassays (CLIA), electrochemiluminescent immunoassays (ECLIA), electrochemical biosensors, and photoelectrochemical biosensors [8,9,10,11]. However, these platforms exhibit significant inter-platform variability, with coefficients of variation (CV) exceeding 20% in multicenter evaluations. For instance, a critical analysis of 500 gastrointestinal malignancy samples revealed platform-dependent detection disparities: the Abbott ARCHITECT i2000SR and Roche cobas e411 immunoassays showed 92.3% concordance in pancreatic adenocarcinoma, but only 36.8% concordance in gastric carcinoma, and ranged from 3.0% to 35.9% in other tumors [12]. This variability poses significant challenges in clinical settings, particularly when considering the expanding utility of CA19-9 in non-gastrointestinal malignancies.

The quantification of CA19-9 exhibits vulnerability to analytical interference from endogenous factors, notably rheumatoid factors (RF), autoantibodies, complement proteins, and heterophilic antibodies (HAs) [13,14,15]. RF, a common autoantibody in patients with rheumatoid arthritis, exhibits nonspecific binding to the Fragment Crystallizable (Fc) portion of immunoglobulins. Autoantibodies targeting self-antigens may cross-react with assay components, while complement proteins activated during inflammation can nonspecifically bind to detection elements, introducing background noise and interference—all of which may contribute to CA19-9 elevation. HAs, capable of binding multiple antigens including those in immunoassays, further induce cross-reactivity and spurious CA19-9 level elevation. The frequency of interference is mainly dependent on the concentration and affinity of the HAs, as well as the detection methods and reagent components used [14].

This study focuses on non-gastrointestinal tumor patients with initial CA19-9 levels exceeding normal ranges detected by Architect/Alinity i systems. We aim to evaluate the consistency between these results and subsequent Elecsys CA19-9 retesting measurements, while systematically analyzing potential factors contributing to CA19-9 elevation.

## 2. Methods

### 2.1. Sample Collection

The study included 5372 non-gastrointestinal tumor patients who underwent CA19-9 testing and their clinical information at the First Affiliated Hospital of Wenzhou Medical University from 8 May 2022–13 February 2023, which was approved from the institutional ethics committee (KY2023-R151). Patients with digestive system diseases were excluded at enrollment. Health screening population must have normal findings on ultrasound, CT, blood tests, and endoscopy (if the patient was tested). After specimen collection, the patient sample must be delivered to the Medical Laboratory Center within 2 h. Centrifuge at 3500 rpm for 8 min was conducted to obtain serum, and testing was performed on the Abbott platform. If elevated CA19-9 levels (>37 U/mL) are detected and inconsistent with the imaging ultrasound results and clinical diagnosis, a repeat test should be conducted on the Roche platform for verification.

### 2.2. CA19-9 Detection Platform

This comparative study employed two automated immunoassay platforms: the ARCHITECT/Alinity i (Abbott Diagnostics, Chicago, IL, USA) utilizing chemiluminescent microparticle immunoassay (CMIA), and the Elecsys CA19-9 (Roche Diagnostics, Basel, BS, Switzerland) implementing electrochemiluminescent immunoassay (ECLIA). Both methodologies target the 1116-NS-19-9 epitope cluster through distinct photonic activation mechanisms. The CMIA platform employs paramagnetic microparticles coated with murine anti-CA19-9 monoclonal antibodies (clone 1116-NS-19-9). Following antigen–antibody complex formation, acridinium-conjugated detection antibodies generate chemiluminescent signals through alkaline peroxide activation, with photon emission at 620 nm quantified via photomultiplier detection. In contrast, the ECLIA system utilizes the tripyridyl-NHS ester-labeled ruthenium (II) antibody forms stable covalent bonds through the reaction of NHS ester with amino groups on the protein, thereby fixing the ruthenium (II)-labeled antibody onto streptavidin. Under controlled potential cycling (0–1.2 V vs. Ag/AgCl), electrochemical excitation induces luminescent complex formation through electron transfer with Tripropylamine co-reactants, achieving detection sensitivity of 0.6 U/mL within 18-min assay duration. Note that the Abbott assay system has a reportable upper limit of 12,000 U/mL. Its actual analytical measurement range is 0–1200 U/mL, with an automatic 10-fold dilution for samples exceeding this threshold. Values above 12,000 U/mL are reported as 12,000 U/mL in our records. Conversely, the Roche assay has an upper detection limit of 1000 U/mL, with values exceeding this threshold truncated to 1000 U/mL.

### 2.3. Interference Exclusion Experiment

Anti-extractable nuclear antigen (ENA) antibody detection: Utilizes the chemiluminescent microparticle immunoassay (CMIA) technology on the MCLIA-800 Plus fully automated multiplex immunoassay analyzer.

Anti-nuclear antibody (ANA) fluorescence detection: Utilizes the Sprinter XL fully automated immunofluorescence system employs HEp-2/primate liver tissue dual-substrate slides.

Rheumatoid factor (RF) and complement testing: Conducted on the Beckman Coulter AU5800 biochemical analysis platform, with RF measured via latex immunoturbidimetry and C3/C4 assays incorporating immunoturbidimetry.

Heterophilic antibody blocking method: This study implemented the HIER-E-001 Nanobody Blocking System (Feipeng Biotech, Guangzhou, China, Lot:20230517]) for HAs interference mitigation. The protocol involved precise 1:1 admixture of blocking reagent with test serum, followed by 15-min isothermal incubation (25 ± 2 °C). Post-incubation specimens underwent centrifugation (10,000× *g*, 10 min) prior to automated immunoassay processing. The HIER-E-001 molecular architecture employs bispecific nanobodies engineered with dual affinity domains: one targeting residual Fc fragments on solid-phase antibodies, the other creating steric hindrance through β-lactoglobulin conjugation.

### 2.4. Statistical Analysis

Given the absence of duplicate serum sample testing, analytical consistency was evaluated through Bland–Altman analysis and Passing–Bablok regression rather than weighted Deming regression (Medcalc [v20.216.7z], Ostend, Belgium). For assessing correlations between CA19-9 elevation rates and (a) distinct clinical comorbidities or (b) varying antigen concentration strata, non-parametric Spearman’s tests were systematically applied. Categorical comparisons of sex-specific CA19-9 elevation patterns across age demographics and disease-associated nonspecific elevation profiles were conducted using Pearson’s chi-square tests (SPSS 23.0, IBM Corp., Armonk, NY, USA).

## 3. Results

### 3.1. Consistency Analysis of ARCHITECT/Alinity I and Elecsys CA19-9 Assays

**Figure 1:**
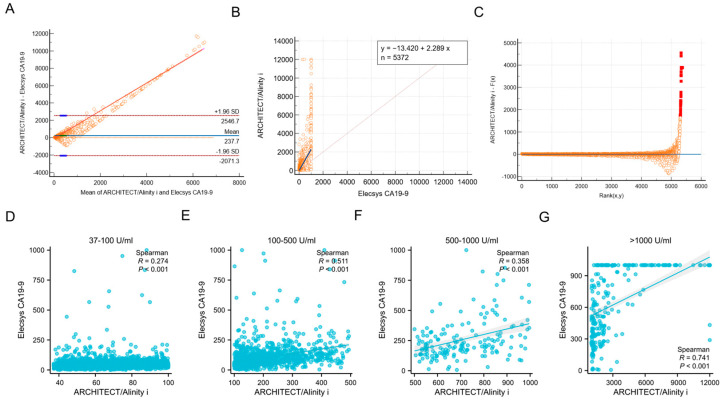
Analysis of the consistency of CA19-9 levels in non-gastrointestinal cancer patients between ARCHITECT/Alinity i and Elecsys CA19-9 assays. (**A**). Bland–Altman analysis of inter-assay agreement between ARCHITECT/Alinity i and Elecsys systems. *X*-axis represents the arithmetic mean of measurements from both assays, while *Y*-axis denotes the inter-method discrepancies. Dashed lines indicate 95% limits of agreement (LOA), solid line denotes mean bias. (**B**,**C**). Passing–Bablok regression between ARCHITECT/Alinity i and Elecsys CA19-9. (**D**–**G**). Correlation of CA19-9 results between architect/alinity i and Elecsys platforms across defined concentration ranges. Note that the Abbott assay system has a reportable upper limit of 12,000 U/mL (actual analytical measurement range: 0–1200 U/mL, with automatic 10-fold dilution for samples exceeding this threshold). Values > 12,000 U/mL are censored to 12,000 U/mL. Conversely, the Roche assay demonstrates an upper detection limit of 1000 U/mL, with values exceeding this threshold truncated to 1000 U/mL.

Among the 5372 samples analyzed, a total of 3470 cases exhibited elevated levels of CA19-9 in both the ARCHITECT/Alinity i (>37 U/mL) and Elecsys CA19-9 (>34 U/mL) assays, yielding a concordance rate of 64.59% (3470/5372). The Bland–Altman bias analysis revealed a mean difference of 237.7, which was markedly distant from the zero-difference line, thereby indicating significant heterogeneity in the consistency between the two assays across different platforms (Figure 1A). The Passing–Bablok regression demonstrated that the CA19-9 test results from Abbott and Roche platforms show significant systematic bias and weak correlation, making the two methods not directly interchangeable (slope = 2.29, 95% CI: [2.206 to 2.375]; intercept = −13.42, 95% CI: [−16.700 to −10.474]) (Figure 1B,C).

Moreover, we stratified CA19-9 results into four tiers: >37–100 U/mL, 101–500 U/mL, 501–1000 U/mL, and >1000 U/mL. The analysis revealed a statistically significant improvement in inter-method concordance when CA19-9 levels exceeded 500 U/mL (Figure 1D–G).

### 3.2. Identification of Endogenous Interfering Substances

**Figure 2:**
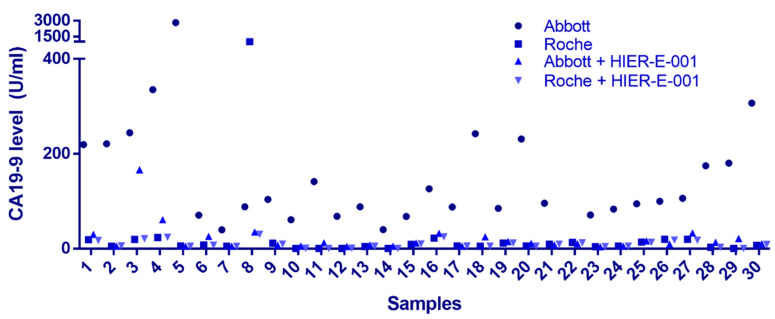
Impact of heterophilic antibody interference on CA19-9 quantification. Solid circles denote CA19-9 concentrations measured by the Abbott assay, while solid squares represent Roche assay results. Upward-pointing triangles indicate Abbott values post-HIER-E-001 treatment, and downward-pointing triangles correspond to Roche values post-HIER-E-001 treatment. *X*-axis: Sample number; *Y*-axis: CA19-9 concentration (U/mL).

Common endogenous interfering substances include RF, autoantibodies, complement, and HAs [16,17]. In our investigation, we randomly selected 30 samples that exhibited a >5-fold disparity in CA19-9 detection results between the two platforms. Upon conducting a comprehensive analysis for these common interfering substances, it was revealed that the levels of RF and complement in these samples were within the normal reference range, with negative results for ANA and ENA antibodies. HAs emerged as a potential candidate for further exploration. Following treatment with the HIER-E-001 blocking reagent, a substantial reduction in CA19-9 levels was observed on the Architect/Alinity i system (*p* < 0.05). Notably, the post-blockade results correlated with the pre-treatment Elecsys CA19-9 measurements in 29 out of 30 cases (96.7%), with the exception of sample 8. These findings collectively suggest that the Architect/Alinity i platform may exhibit a heightened vulnerability to HAs interference in the context of non-gastrointestinal malignancies (Figure 2). In the case of sample 8, the initial testing results of the Elecsys detection system were also impacted, indicating that the specific heterophilic antibody in question may possess the capacity to influence the Elecsys platform as well. For sample 3, the reduction after treatment with blocking reagent was not sufficient. This may be due to the limited effectiveness of currently used allergen-specific blocking reagents in clinical practice for blocking high-affinity HAs or certain antibody subclasses. If the sample contains an excessively high concentration of HAs or exhibits abnormal affinity, residual interference may remain.

### 3.3. Compliance Analysis of Results with Clinical Diagnosis

**Figure 3:**
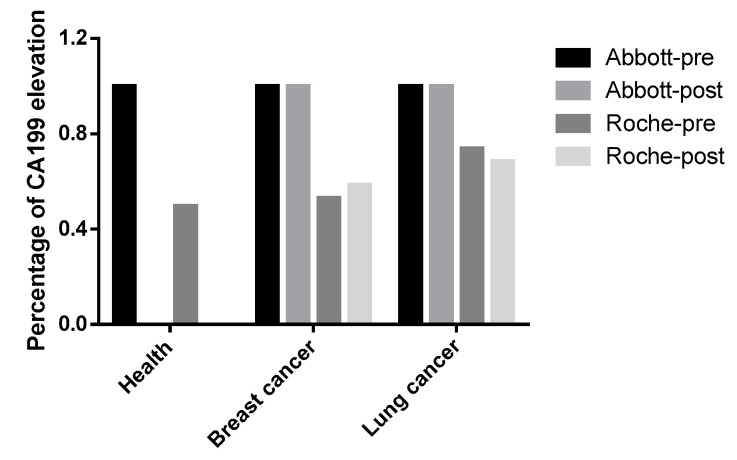
Comparative Analysis of CA199 Elevation Patterns in Healthy Cohort, Lung and Breast Cancer. Differential performance of Abbott and Roche diagnostic systems in detecting CA199 elevation across healthy, lung and breast cancer cohorts, with pre- and post-intervention comparisons.

Among 985 health checkup population exhibiting ARCHITECT/Alinity i-detected CA19-9 elevations (presumed false positives in non-oncological contexts), Elecsys confirmed detectable elevations in only 49.64% (489/985). This observation warrants re-evaluating whether the application of CA19-9 testing in healthy populations is clinically warranted. Moreover, our findings revealed that non-specific CA19-9 elevation was frequently observed in lung and breast cancers, which is consistent with previous clinical studies [18,19]. Further stratification into preoperative and postoperative subgroups was conducted for additional analysis. For the breast cancer cohort, the preoperative subgroup (*n* = 34): Elecsys identified nonspecific CA19-9 elevations in 52.94% (18/34). The postoperative subgroup (*n* = 123): detection rate increased to 58.54% (72/123). For the lung cancer cohort, the preoperative subgroup (*n* = 103): Elecsys detected elevated CA19-9 in 73.79% (76/103). The postoperative subgroup (*n* = 57): elevations were observed in 68.42% (39/57). CA19-9 has potential value as a tumor biomarker for lung and breast cancer. However, since our samples were not pre- and postoperative pairs from the same patients, the postoperative group of lung and breast cancer patients did not show a significant decrease in CA19-9 levels (Figure 3). Notably, critical limitations of this study must be acknowledged, including selection bias inherent to the exclusive inclusion of ARCHITECT/Alinity i-positive cases, which inherently restricts generalizability to assay-negative populations.

### 3.4. Assessment of Disease Risk Factors for Non-Specific Elevated CA19-9

**Figure 4:**
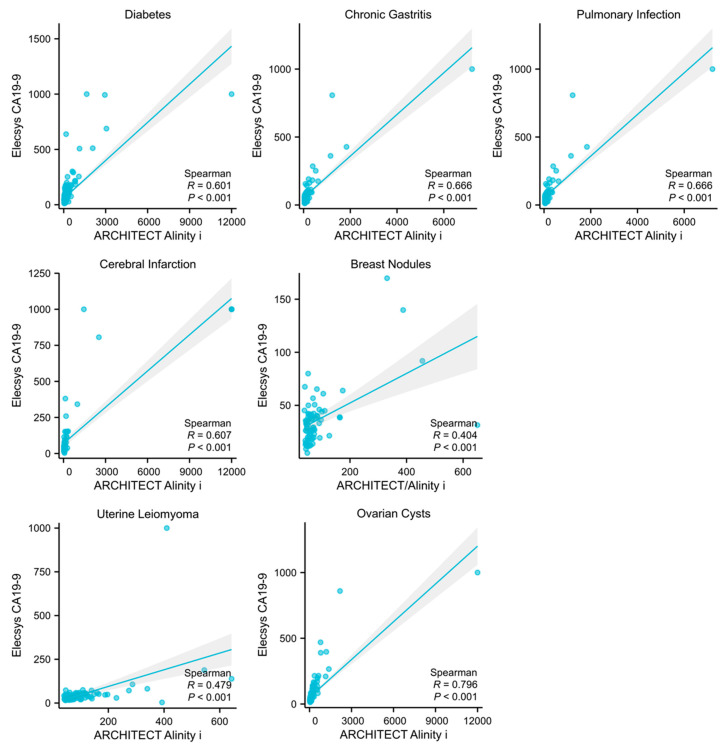
Correlation analysis of the detection results in different underlying diseases. Figures represent the correlation levels of two assays in detecting CA19-9 in subgroups of diabetes, chronic gastritis, pulmonary infection, breast nodules, uterine leiomyoma, and ovarian cysts, respectively.

Among the 5372 cases, subgroups with a sample size exceeding 40 included individuals with the following underlying conditions: diabetes (*n* = 258), cerebral infarction (*n* = 81), chronic gastritis (*n* = 122), pulmonary infection (*n* = 90), ovarian cysts (*n* = 144), breast nodules (*n* = 71), and uterine leiomyoma (*n* = 113). Patients were subsequently stratified into CA19-9 elevated and non-elevated groups based on their specific underlying diseases. Elevated levels in both assays were categorized into the elevated group. Upon analysis, it was determined that diabetes (*p* = 0.0005, 95% CI: 1.252–2.230), pulmonary infection (*p* = 0.0017, 95% CI: 1.384–4.003), breast nodules (*p* = 0.0017, 95% CI: 0.291–0.745) and uterine leiomyoma (*p* = 0.0017, 95% CI: 0.375–0.790) were significant factors contributing to the non-specific elevation of CA19-9 (Table 1). Furthermore, a linear regression analysis was conducted on the CA19-9 results from both assays among patients with different underlying diseases. This analysis revealed a moderate yet statistically significant correlation in the results for the diabetes, chronic gastritis, pulmonary infection, cerebral infarction, and ovarian cysts subgroups (Figure 4).

### 3.5. Correlation Between Non-Specific Elevation of CA19-9 with Age

**Figure 5:**
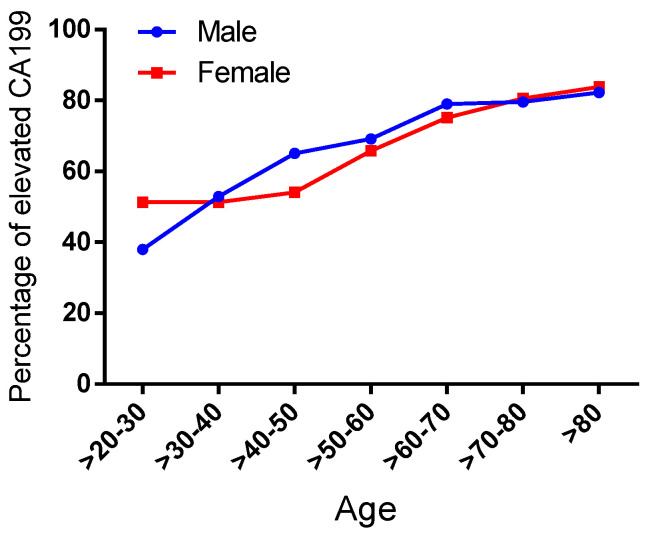
The detection rate of elevated CA19-9 varies among different age and sex groups. The *x*-axis represents different age groups, and the *y*-axis represents the detection rate. The blue curve represents males, and the red curve represents females.

Given that CA19-9 has been confirmed to correlate with non-gastrointestinal tumor and inflammatory etiologies, we proposed a retrospective investigation into the gender-related and age-dependent patterns of its non-specific elevation. In the analysis of 5372 cases with dual-assay-validated CA19-9 measurements, gender stratification revealed a statistically significant predominance of non-specific elevation in females compared to males (61.72% vs. 71.16%, *p* < 0.0001), suggesting potential hormonal regulatory mechanisms (Table 2). For age analysis, we divided the cases into seven age intervals: >20–30, >30–40, >40–50, >50–60, >60–70, >70–80, and >80 years. Age-dependent analysis across seven intervals (>20–30 to >80 years) demonstrated progressive escalation in both cohorts. In the male subgroup, the proportions of CA19-9 elevation in these intervals were 37.97%, 52.87%, 65.04%, 69.11%, 78.97%, 79.51%, and 82.23%, respectively. For the female subgroup, the proportions of non-specific CA19-9 elevation in these intervals were 51.30%, 51.28%, 54.00%, 65.77%, 75.12%, 80.58%, and 83.81%, respectively (Figure 5 and Table 3). Notably, within the reproductive-age cohort (21–30 years), female subjects exhibited a 51.30% prevalence of non-specific CA19-9 elevation, demonstrating a statistically significant disparity (*p* < 0.001) compared to the 37.97% observed in age-matched males. This difference may be related to the physiologic elevation of CA19-9 during pregnancy [20].

## 4. Discussions

This study analyzed the performance of CA19-9 detection across Architect/Alinity i and Elecsys platforms in a large cohort of non-gastrointestinal malignancies, revealing the clinical impacts of methodological variances, endogenous interference, underlying diseases, gender, and age on nonspecific CA19-9 elevation. Our findings highlight the complexity of interpreting CA19-9 levels in non-gastrointestinal clinical settings, providing critical insights for result interpretation in both routine health checkup populations and patients with prior medical conditions undergoing cancer screening.

In non-gastrointestinal malignancies, the inter-platform concordance rate between the two assays was 64.59% (3470/5372). This platform-dependent variability raises concerns regarding result interchangeability in clinical practice. Such methodological inconsistency persists even in gastrointestinal malignancies [21,22,23]. A study using 135 pancreatic cancer samples compared five CA19-9 detection assays (ADVIA Centaur, ARCHITECT i2000, UniCel DxI 800, IMMULITE 2000, and Elecsys E170), with ADVIA Centaur as the reference method. The correlation coefficients were 0.98 (ARCHITECT i2000), 0.98 (UniCel DxI 800), 0.87 (IMMULITE 2000), and 0.85 (Elecsys E170) [21]. The observed variability in carbohydrate antigen quantification across diagnostic platforms arises from two fundamental limitations: (1) The lack of unified national or international standards and reference measurement for glycoprotein tumor marker assays prevents standardized metrological traceability between detection platforms, (2) technical divergences in antibody epitope recognition patterns, calibrator composition, and interference resistance collectively drive inter-system discrepancies. These factors ultimately pose unreliable cross-platform comparisons in clinical decision-making. The same detection assay for CA19-9 must be consistently used during dynamic disease monitoring, with the specific analytical platform explicitly stated in the report. Clinicians are urged to analyze the CA19-9 interpretation thoroughly and consider changing the test platform when CA19-9 levels do not match the clinical diagnosis [12,21].

The established interference mechanisms of HAs in immunoassays primarily manifest in both sandwich assays and competitive immunoassays [24]. On the ARCHITECT/Alinity i platform, 96.67% (29/30) of discordant samples normalized post-blockade, establishing HAs as the dominant interference source in non-gastrointestinal malignancies. Among the 30 cases we selected, 29 exhibited interference from HAs, likely due to the random selection of specimens and the fact that other factors besides HAs had not as significant an impact on the pseudoelevation of CA19-9 levels. Notably, Sample 8 demonstrated Elecsys result perturbation, indicating the presence of cross-reactive HAs capable of binding shared epitopes across both platforms. Currently, most antibodies used in clinical immunoassays are derived from laboratory animals. Heterophilic antibodies, characterized by multispecific binding properties, can interact with antibodies from multiple animal species used in reagent preparation. Through bridging capture antibodies, labeled antibodies, or labeled antigens, HAs interfere with detection systems, leading to nonspecific positive or false-negative results [25,26].

The CA 19-9 tumor marker test is widely used for screening purposes, and clinical practice frequently encounters asymptomatic patients with elevated CA 19-9 levels. However, the clinical utility of CA 19-9 tumor marker screening in health checkup population remains undetermined [27]. In the health screening cohort with elevated CA19-9 levels detected on the Abbott platform, we concurrently observed 49.64% elevation on the Roche platform, confirming the existence of non-specific CA19-9 elevation in healthy individuals. It must be emphasized that while these subjects showed no clinical symptoms or abnormalities in routine examinations (including CT, ultrasound, and blood tests), the potential presence of occult pathologies cannot be entirely excluded.

We found non-specific CA 19-9 elevation in association with diabetes, pulmonary infections, breast nodules, and uterine leiomyomas, suggesting that CA 19-9 levels may be elevated in benign conditions, and that it is important to be aware of pseudo-elevations and clinical interpretations of CA 19-9 levels when measuring them in asymptomatic populations of patients. When interpreting CA19-9 results in patients with benign conditions, clinicians must systematically account for diabetes, pulmonary infections, and gynecological conditions [19,28,29,30]. The sex-specific elevation pattern and age-dependent trends across both cohorts reveal multifactorial regulation of CA19-9 levels. This finding aligns with current literature reports [31,32]. The physiologic elevation of CA19-9 that occurs in women of childbearing age tends to decline in mid- to late pregnancy [20]. Age-related increases in CA19-9 may be associated with cumulative mucosal damage, chronic inflammation, and aging. Although these elevations demonstrate limited oncological specificity, their persistent manifestation retains significance in clinical evaluation. This finding aligns with current research, demonstrating that CA 19-9 levels may be elevated across various benign diseases, and that CA 19-9 measurement for malignancy screening in asymptomatic patients lacks clinical practicality [33].

The epitope of CA19-9 primarily consists of the sialyl-Lewis A structure, but in practice, multiple analogous glycan structures exist, such as sialyl-Lewis C and variants containing N-glycolylneuraminic acid (Neu5Gc). These structures exhibit high similarity to sialyl-Lewis A in both chemical composition and spatial conformation, which may lead to antibody recognition cross-reactivity. For instance, certain monoclonal antibodies (e.g., AB1 and AB5) can recognize both sialyl-Lewis A and sialyl-Lewis C, whereas AB2 demonstrates high specificity for sialyl-Lewis A [34]. This glycan heterogeneity complicates detection and may contribute to discrepancies across different assay platforms. Moreover, metabolic dysregulation and microenvironmental changes in tumor cells can alter CA19-9-associated glycan modifications, generating further structural isoforms. For example, in pancreatic ductal adenocarcinoma (PDAC), spatial variations in CA19-9 and CD175 (Tn antigen) expression have been observed: CA19-9 is predominantly expressed on O-glycans, while CD15s (sialyl-Lewis X) localizes to both N-glycans and O-glycans [35]. This differential expression suggests that tumor cells may express unique glycosyltransferases that influence the glycan structures of CA19-9, potentially modifying its antigenicity and detection characteristics.

The specificity and affinity of monoclonal antibodies are critical determinants of CA19-9 assay accuracy. Significant variations exist in epitope specificity among different monoclonal antibodies targeting CA19-9. For instance, the novel antibody RA9-23, engineered via yeast surface display (YSD) platform evolution, exhibits stronger binding capacity with higher affinity (KD = 9–37 nM) compared to native antibodies (KD = 42 nM) [4]. Such affinity disparities may lead to differential sensitivity among antibodies when detecting low CA19-9 concentrations. Additionally, antibody specificity significantly impacts detection outcomes. For example, certain antibodies (e.g., AB1 and AB5) can recognize both sialyl-Lewis A and sialyl-Lewis C, whereas AB2 exhibits high specificity for sialyl-Lewis A [34]. Such specificity differences may lead to cross-reactivity across distinct detection platforms. For instance, the GCTM-5 antibody, while partially reactive with CA19-9 antibodies, demonstrates divergent reactivity in glycan array analyses compared to sialyl-Lewis A [36]. Variations in assay platform design also influence CA19-9 detection results. Differences among platforms (e.g., ELISA, chemiluminescent immunoassays) in antibody immobilization, signal amplification, and detection conditions may enhance sensitivity toward specific glycan structures, thereby generating platform-dependent cross-reactivity. For example, some platforms may exhibit heightened sensitivity to non-human-synthesized glycan structures like Neu5Gc, potentially leading to false-positive results [34].

Based on this study, when performing CA19-9 testing, clinicians should critically assess the necessity of ordering this test for asymptomatic individuals. If results are inconsistent with clinical diagnoses, comprehensive evaluation must include pre-analytical, analytical, and post-analytical quality control, patients’ underlying conditions, and heterophilic antibody interference—with particular attention to women of reproductive age and patients with autoimmune comorbidities [37,38,39].

While this study provides robust evidence of methodological differences between the ARCHITECT/Alinity i and Elecsys platforms, the exclusive inclusion of cases with ARCHITECT/Alinity i-elevated CA19-9 levels introduced selection bias by omitting negative cohorts, potentially inflating the observed discordance rate. Future multicenter investigations should incorporate both positive and negative detection cohorts for comprehensive comparative analysis, enabling the establishment of validated platform-specific reference ranges. The development of next-generation CA19-9 assays requires the integration of interference-specific epitope engineering and glycosylation-specific analytical capabilities, which is a critical unmet need in precision oncology.

## Figures and Tables

**Table 1:** Univariate analysis of different underlying disease on elevated CA19-9 levels.

Disease	Number	χ^2^ Value	*p*-Value	Odds Ratio (95% CI)
Diabetes	257	11.96	0.0005	1.671 (1.252–2.230)
Cerebral Infarction	81	2.685	0.101	1.506 (0.920–2.465)
Chronic Gastritis	122	0.130	0.719	1.072 (0.733–1.568)
Pulmonary Infection	90	9.886	0.0017	2.354 (1.384–4.003)
Breast Nodules	71	9.858	0.0017	0.466 (0.291–0.745)
Ovarian Cysts	144	0.036	0.845	1.052 (0.742–1.492)
Uterine Leiomyoma	113	9.883	0.0017	0.544 (0.375–0.790)

**Table 2:** The proportion of elevated CA19-9 levels among different genders.

Gender	Positive Count	Total	Positive Rate
Male	1419	1994	71.16%
Female	2051	3378	61.72%
Total	3470	5372	
χ^2^ value			59.38
*p*-value			<0.0001

**Table 3:** The proportion of elevated CA19-9 levels among different genders across various age groups.

Age	Male Positive Rate	Female Positive Rate	χ^2^ Value	*p*-Value
0–20	58.82% (10/17)	46.15% (36/78)	0.190	0.663
>20–30	37.97% (30/79)	51.30% (198/386)	4.656	0.031
>30–40	52.87% (92/174)	51.28% (321/626)	0.139	0.710
>40–50	65.04% (173/266)	54.00% (466/863)	10.090	0.002
>50–60	69.11% (311/450)	65.77% (442/672)	1.360	0.244
>60–70	78.97% (383/485)	75.12% (305/406)	1.858	0.173
>70–80	79.51% (295/371)	80.58% (195/242)	0.103	0.748
>80	82.23% (125/152)	83.81% (88/105)	0.108	0.742

## Data Availability

Data will be made available on request.
